# Architecture‐Engineered Semi‐Crystalline Microporous Polymer Membranes for Precise Molecular Separation

**DOI:** 10.1002/advs.77001

**Published:** 2026-08-05

**Authors:** Zi‐Meng Xu, Hao Zhuo, Song‐Hui Zhi, Xiao‐Feng Zhong, Pan‐Pan Zhang, Si‐Yuan Yang, Wen‐Yong Yang, Guang‐Yong Shen, Ya‐Zhao Li, Yi Li, Ming Xue

**Affiliations:** ^1^ School of Chemical Engineering and Technology School of Chemistry Southern Marine Science and Engineering Guangdong Laboratory (Zhuhai) OFMMT Sun Yat‐sen University Guangzhou People's Republic of China; ^2^ China Lucky Group Corporation Baoding People's Republic of China

**Keywords:** microporous membranes, organic solvent nanofiltration, semi‐crystalline polymers, separation

## Abstract

Amorphous polymer membranes typically suffer from low fractional free volume (FFV) due to structural flexibility, limiting separation performance and operational stability. Herein, we propose a strategy that constrains segmental motion by *π*–*π*‐driven ordered stacking. Guided by Random Forest (RF) analysis of substructure marginal contributions, we selected a pre‐existing high FFV aromatic substructure (*p*‐terphenyl) and, inspired by articular joints, assembled it into rigid *π*–*π*‐stacked domains linked by flexible amide units, creating a semi‐crystalline membrane (DATP) characterized by short‐range order embedded within long‐range disorder. Organic‐organic interfacial polymerization (OOIP) yields this scalable network, further reducing transport resistance by tuning ordered and disordered domain arrangements. The DATP membrane features rigidly interconnected channels, increasing FFV by 14% to 35.5% compared to the amorphous membrane (DAB), while reducing interconnected volume loss during operation by 14.3%. This achieves a record‐high methanol permeability of 2779 L m^−2^ h^−1^ bar^−1^ nm and the highest selectivity among all polymer membranes with comparable molecular weight cut‐off (MWCO). Extended to active pharmaceutical ingredients (APIs) separation, it enables 50‐fold enrichment within 2.5 h and shows 28% higher selectivity than commercial membranes. By endowing polymer membranes with the structural tunability of crystalline materials, this work combines well‐defined pore architecture with scalable fabrication.

## Graphical abstract

Guided by a Random Forest model, a *π*–*π*‐driven ordered stacking strategy deploys functionally distinct substructures to restrict chain segment motion and increase free volume, while preserving the intermolecular interactions that maintain structural integrity. The resulting microporous semi‐crystalline polymer membrane features rigid, low‐resistance, interconnected transport channels, overcomes the permeability‐selectivity trade‐off, and combines high‐performance separation with scalable fabrication.

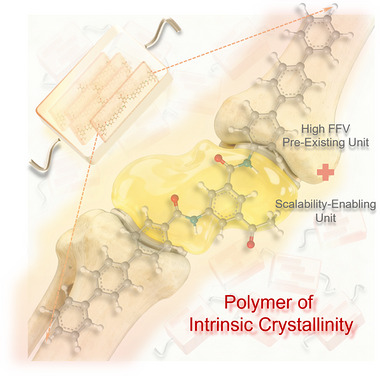

## 1 Introduction

Membrane separation represents a promising technology for the global transition toward a net‐zero future, owing to its lower energy consumption and carbon emissions [1, 2]. The core of this process relies on membrane materials enabling precise molecular separation with low energy input [3, 4, 5]. The scalability of amorphous polymer membranes is a key advantage, but their performance is constrained by the trade‐off between permeability and selectivity. This limitation originates from their lack of interconnected pores and a broad distribution of pore sizes [6, 7]. In contrast, crystalline porous materials such as metal‐organic frameworks (MOFs), with their highly ordered structures and abundant microporosity, offer promising opportunities for efficient molecular sieving [8, 9]. However, issues during synthesis, namely intercrystallite defects and lattice cracks, have limited the fabrication of crystalline MOF membranes to less than 1% of known MOF structures [10, 11]. A moderation strategy involves combining the scalability of polymers with the well‐defined pore architectures of crystalline materials, as in mixed‐matrix membranes (MMMs) prepared via solution blending [12]. Nevertheless, poor compatibility between polymers and fillers, along with challenges in reducing membrane thickness to the nanoscale, often leads to performance that falls short of theoretical predictions [13, 14]. Therefore, engineering materials that simultaneously exhibit high permeability, high selectivity, and scalability present a formidable challenge, but it is urgently needed.

We aimed to increase fractional free volume (FFV) while constraining chain segment motion, which requires substructures with high persistence length in the polymer backbone. Various strategies that assign restrictive functions to linking bonds have been explored [15, 16], for example, introducing alkynyl groups (–C≡C─) via Sonogashira‐Hagihara coupling. These rigid linkages with low conformational freedom suppress dense chain packing and prevent structural relaxation by restricting segment bending and rotation [17, 18]. However, highly crosslinked rigid conjugated frameworks resist conventional solution processing, and reliance on precious metal catalysts limits scalability [19]. We therefore propose a strategy that exploits intermolecular interactions to drive ordered stacking to constrain chain segment motion. The polymer backbone incorporates short‐range order within long‐range disorder, combining the structural tunability of crystalline materials with the inherent processability of polymers. Nevertheless, a multiscale contradiction remains. Aromatic rings strongly enhance FFV, but larger rings introduce excessively strong intermolecular interactions (e.g., *π–* *π* stacking), which tighten chain packing and reduce FFV [20, 21, 22]. This reveals the central dilemma: how to precisely select monomer substructures that increase FFV without weakening the intermolecular interactions essential for structural persistence, and how to assemble them using linkages amenable to scalable fabrication.

Inspired by articular joints, where rigid backbones are connected by flexible articulations, we designed a structure in which ordered rigid domains define transport pathways by framing the disordered regions between them. *p*‐Terphenyl, an aromatic unit capable of *π‐*‐*π* stacking, is selected as the substructure to pre‐establish and sustain high FFV. The rationale for this choice is supported by the marginal contributions of aromatic‐ring‐containing substructures, as predicted by the Random Forest (RF) model. To further interconnect these ordered domains and extend them into multidimensional networks, we employ interfacial polymerization (IP) to incorporate flexible amide linkages between aromatic units. The incorporation of these bonds decouples the rigid connection between ordered domains, while their conformational flexibility suppresses excessive intermolecular interactions. This mechanism prevents defect formation by mitigating stress concentration and, owing to the mild reaction conditions, ensures the scalability of the membrane. Furthermore, this fabrication method enables simultaneous control over the arrangement of both ordered and disordered domains, further reducing transport resistance. Specifically, the addition of tetrahydrofuran (THF) to the aqueous phase accelerates the assembly kinetics of ordered domains, inducing a metastable edge‑on stacking configuration in which segments align perpendicular to the substrate. For the disordered domains, the reduced interfacial tension promotes the diffusion of amine monomers into the organic phase, thereby breaking the dimensional confinement of the reaction. By synergistically integrating the constraints imposed by intermolecular interactions with the advantages of interfacial polymerization, we rationally selected and assembled distinct functional substructures to construct a low‐resistance semi‐crystalline polymer membrane. This strategy enhances permeability while improving selectivity, achieving a synergistic combination of well‑defined pore architecture and scalable fabrication for mass transfer intensification in precise molecular separation.

## 2 Results and Discussion

### 2.1 Characterization and Properties of Semi‐Crystalline Microporous Polymer Membranes

The free volumes in microporous polymer frameworks arise from the contributions of their rigid and contorted backbone structures or bulky monomer units, which hinder efficient packing. Enhancing FFV while preserving structural stability requires balancing FFV and intermolecular interactions, under an additional processability constraint. An optimal *π*‐stacking unit should provide sufficient *π*—conjugated surface, a rigid and directional geometry that favors ordered packing, and a topology that avoids excessive face‐to‐face aggregation. It must also remain synthetically accessible and solution‐processable, without compromising solubility or shifting pore size distribution outside the nanofiltration range. Relative to benzene and biphenyl, *p*‐terphenyl offers a larger *π*‐system and a linear, rigid backbone, while avoiding the strong aggregation and poor processability often associated with larger fused polyaromatics. Moreover, steric repulsion between ortho hydrogens on adjacent phenyl rings induces a non‐coplanar conformation, which helps suppress overstacking [16]. We therefore selected the *p*‐terphenyl unit to enhance the FFV while preserving the intermolecular interactions essential for structural persistence. Furthermore, constructing a framework amenable to multidimensional extension requires mild and rapid bond‐forming chemistry [17]. Taking inspiration from articular joints, we introduced freely rotating amide bonds via room‐temperature interfacial polymerization to serve as flexible “ligaments” connecting rigid “joints” (*p*‐terphenyl stacking units), thus creating interconnected transport pathways. Consequently, the pre‐existing high‐FFV rigid aromatic monomer 4,4’‐diamino‐p‐terphenyl (DATP) and the trimesoyl chloride (TMC) that enable multidimensional scalability were chosen to fabricate the polyamide (PA) membrane (Figure S1). To probe how substructures affect FFV and intermolecular interactions, *p*‑phenylenediamine (PPD) and 4,4’‐diaminobiphenyl (DAB) were used as controls. Because the amine monomer is the only variable, each membrane is named after its corresponding amine abbreviation.

**FIGURE 1:**
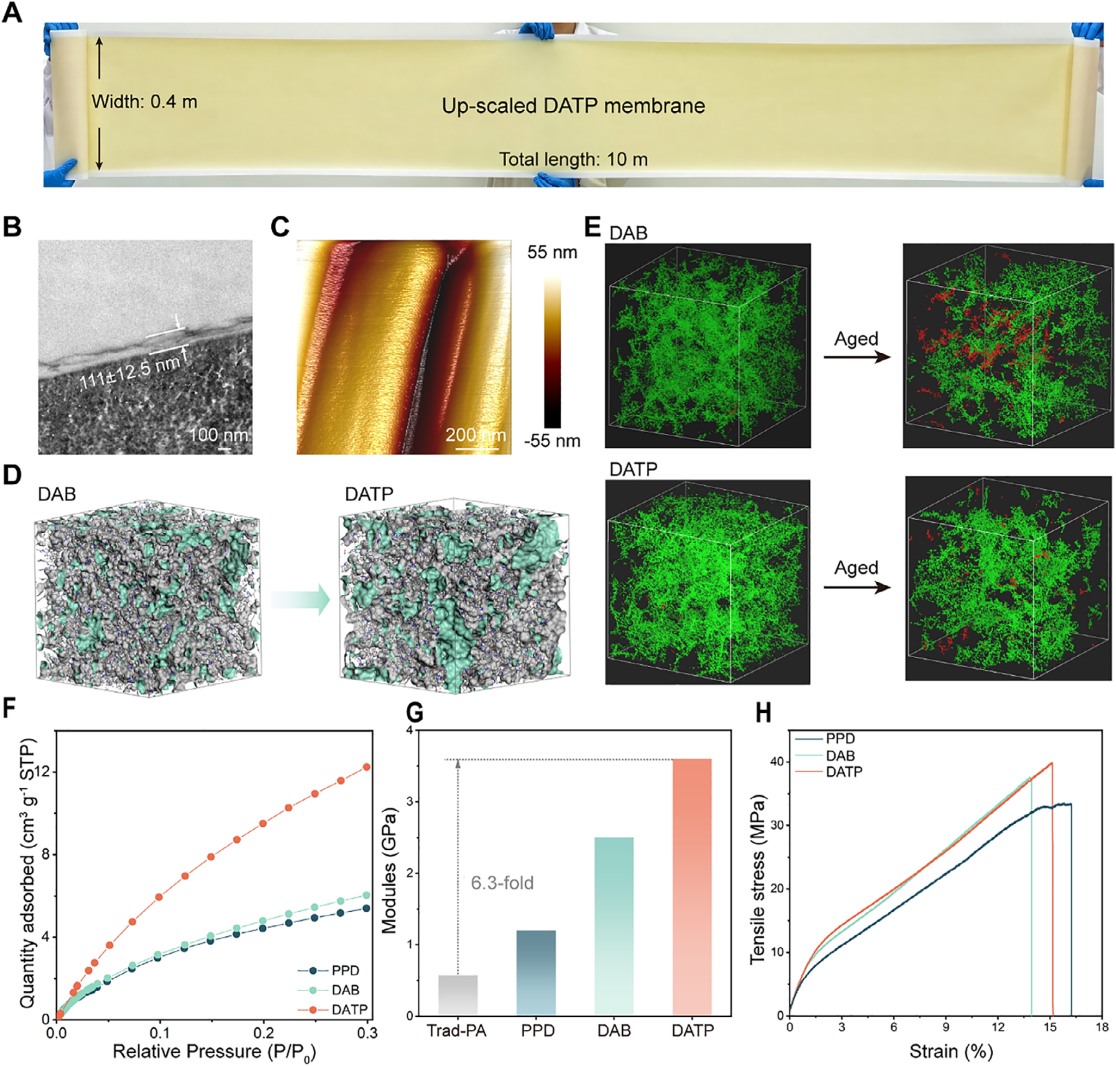
Morphology and characteristics of semi‐crystalline microporous polymer membranes. (A) Photograph of a large‐scale DATP membrane. (B) Transmission electron microscopy (TEM) images of the DATP membrane. (C) Surface atomic force microscopy (AFM) images of the DATP membrane. (D) Simulated energy‐minimized 3D amorphous cells of DAB and DATP networks, accessible free volume at a probe radius of 0.5 Å. (E) 3D view of the cells of DAB and DATP membranes (left). Images of aged cells (right). Red shading represents isolated free‐volume elements and green shading represents interconnected micropores. (F) CO_2_ uptakes of three membrane powders at 273 K. (G) Young's modulus histogram of three membranes. (H) Stress‐strain curves of the three membranes tested using a microcomputer‐controlled electronic tensile testing machine.

The DATP membrane, successfully synthesized via in situ interfacial polymerization on a crosslinked polyimide (P84) support, is confirmed by Fourier‐transform infrared (FT‐IR) spectroscopy and solid‐state ^13^C nuclear magnetic resonance (NMR) spectroscopy (Figures S2 and S3), and exhibits intrinsic scalability (Figure 1A and Figures S4–S7). Characterized by a laminar structure and a thickness of approximately 120 nm, the DATP layer displays greater surface roughness (*R*_a_ = 17.2 nm) than control membranes, which translates to an enlarged effective filtration area (Figure 1B,C and Figures S8–S10) [23]. Molecular simulations of three membrane structures show that the rigid‐monomer‐based DATP membrane exhibits approximately 14% and 35.5% greater cumulative pore volume than PPD and DAB membranes, respectively (Figure 1D and Figures S11–S13). Considering the possible swelling in organic solvents and the presence of motion‐enabled regions under applied pressure, we performed force‐field aging of three membrane structures in methanol. After the treatment, the DATP membrane cell model retained the most interconnected volume elements, losing approximately 14.3% less interconnected volume elements than the DAB membrane — evidence of its superior rigidity and anti‐swelling capacity (Figure 1E and Figure S14). To circumvent the limitations of N_2_ at 77 K, namely diffusion restrictions, quadrupole‐specific adsorption, and insufficient resolution for ultramicropores (<0.7 nm), CO_2_ was selected as the probe molecule for accurate characterization of these membranes [24]. The enhanced CO_2_ uptake of the DATP membrane relative to DAB and PPD membranes at low pressure and 273 K validates the substantial increase in FFV (Figure 1F and Figure S15). Notably, the DATP membrane exhibits a Young's modulus of 3.6 GPa — a 6.3‐fold increase over the traditional PA membrane (Figure 1G and Figure S16) [17]. This is consistent with its higher tensile strength and reduced elongation at break (Figure 1H).

### 2.2 Architectural Engineering of Semi‐Crystalline Microporous Polymer Membranes

**FIGURE 2:**
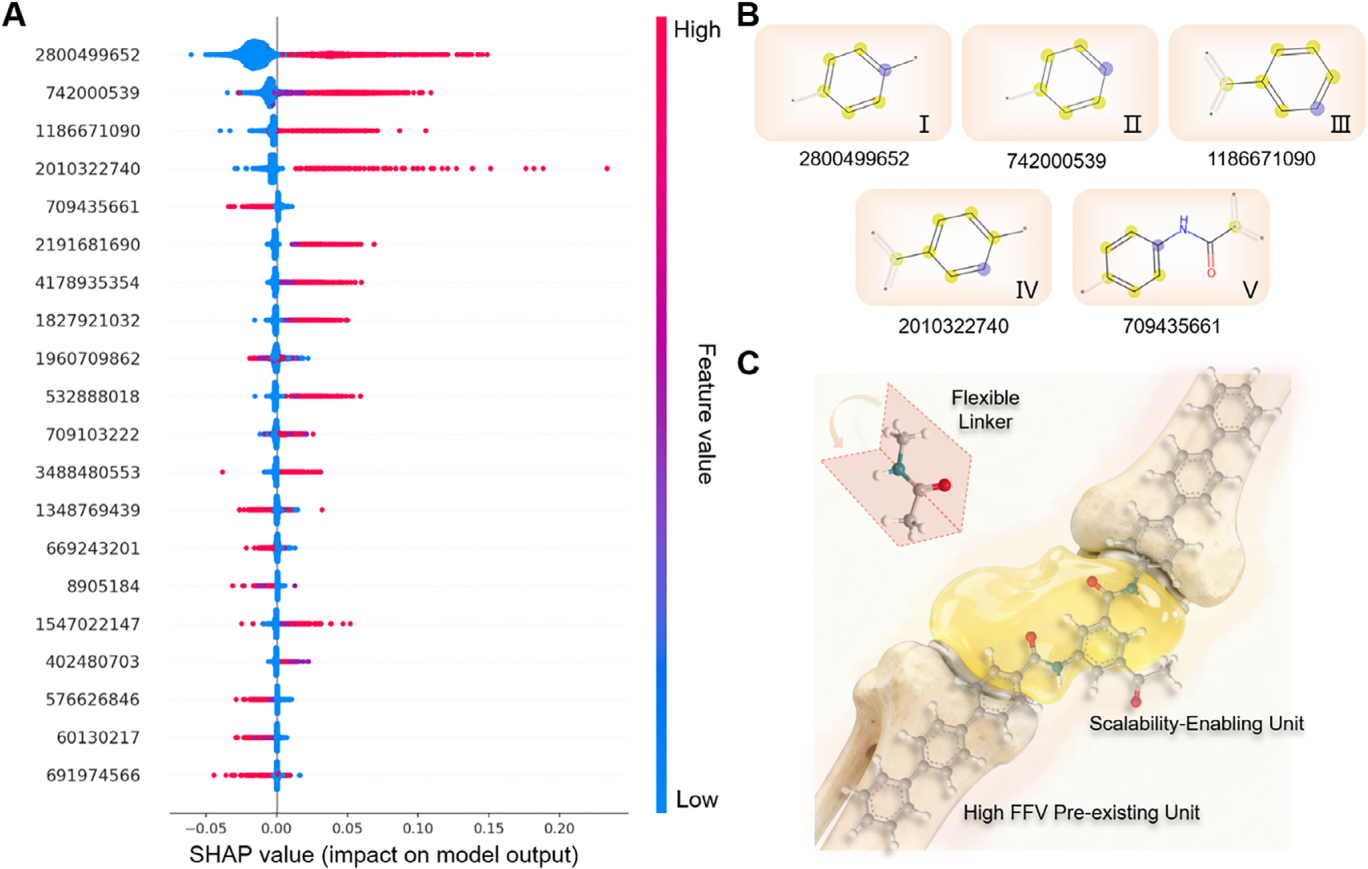
Impact of the aromatic‐ring‐containing substructural features on fractional free volume. (A) Results of SHAP analysis based on the RF models. (B) Five substructures containing aromatic rings with significant impact on FFV. (C) Design concept for readily extensible semi‐crystalline microporous polymer membranes with high pre‐existing FFV.

Quantifying the contribution of aromatic ring substructures to polymer FFV is key to rationalizing the DATP monomer selection. We therefore employed machine learning (ML) to capture the complex nonlinear relationships between substructural features and FFV. The models were trained on polymer chemistry data from the PoLyInfo database. Polymer repeating units were converted to SMILES strings and characterized by their Morgan Fingerprint Frequency (MFF) (Figure S17) [25]. These fingerprint vectors were then analyzed to identify common substructures. Considering that aromatic ring units are universally employed in the construction of substructures, we applied the Random Forest (RF) model (Figure S18) [22], utilizing Shapley Additive exPlanations (SHAP) to quantitatively deconvolve the impact of these substructures on the FFV (Figures S19–S22). Figure 2A presents the SHAP analysis for the twenty most influential substructures containing aromatic rings. Among these, solid lines denote C─C bonds within an aromatic ring. Substructure aromatic ring II exerts a strong positive influence on FFV (Figure 2B), as evidenced by the correlation between high feature values and positive SHAP scores. This indicates that the outward extension of phenyl rings via single bonds is a key structural determinant for enhancing FFV. Building on this, the substantial positive contributions also observed from substructure aromatic rings I and III lead us to infer that para‐conjugated linkages between phenyl rings are particularly advantageous. This inference is definitively corroborated by the highest SHAP value exhibited by substructure aromatic ring IV, revealing that the most favorable structural unit requires a conjugated backbone comprising no fewer than three phenyl rings. This configuration arises from two specific factors: the planar rigidity of aromatic rings constrains backbone conformational freedom, while the para‐linkage maximizes steric hindrance to disrupt efficient chain packing. Consequently, the non‐equilibrium, high‐free‐energy state is generated during membrane formation and characterized by enhanced free volume. This state becomes kinetically trapped, thereby inhibiting structural relaxation toward thermodynamic equilibrium [26]. Meanwhile, the negative influence associated with the amide bond in aromatic ring V is deemed unavoidable, owing to the tendency of highly mobile flexible segments to achieve higher packing density in the equilibrium state, as the denser packing minimizes the free energy of the system. This implies that three reactive crosslinking sites were placed at the meta‐positions of the phenyl rings, a design that effectively minimizes the loss of FFV upon network formation (Figure 2C).

**FIGURE 3:**
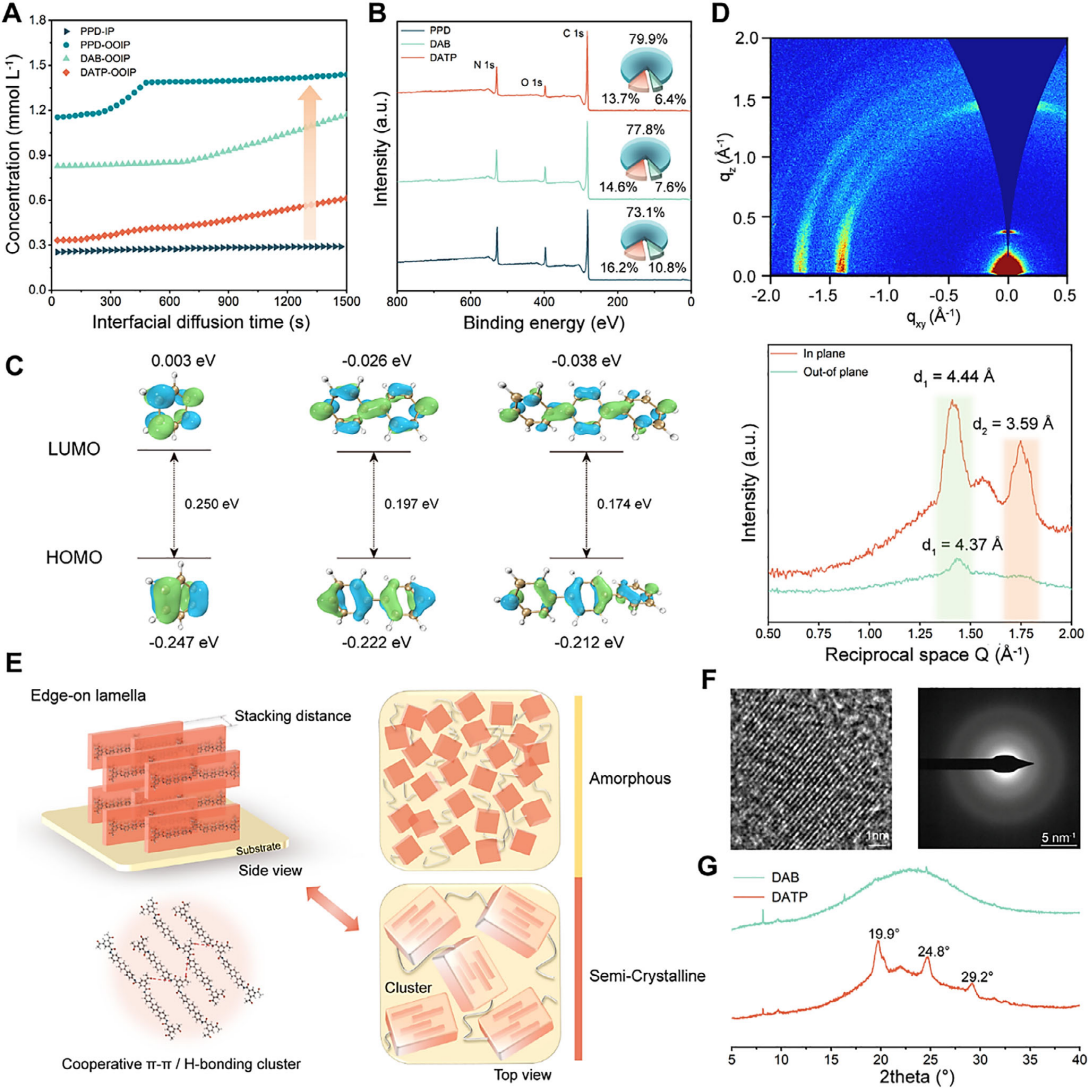
Physicochemical properties of semi‐crystalline microporous polymer membranes. (A) Amine monomer concentrations in the *n*‐hexane phase over time. (B) XPS spectra of three membranes. Elemental compositions are presented in the pie chart below. Atomic color codes: C: cyan, O: orange, N: green. (C) HOMO–LUMO distributions of the aromatic units, with their corresponding energy gaps (*E*_gap_). (D) 2D and 1D GI‐WAXS images of DATP nanofilm, where *Q*_xy_ and *Q*_z_ denote the reciprocal space parallel (xy) and perpendicular (z) to the nanofilm surface, respectively. (E) Schematic illustrations of edge‐on orientation semi‐crystalline and amorphous polymer membranes. (F) High‐resolution TEM image and corresponding electron diffraction pattern of DATP membrane. (G) XRD patterns of DATP and DAB membranes.

The organic‐organic interfacial polymerization (OOIP) strategy affords an additional advantage in the synthesis of the DATP membrane (Figure S23), namely the synergistic control over the arrangement of both disordered and ordered domains. Central to this strategy is the introduction of THF into the aqueous phase. To regulate the disordered domains, THF both solubilizes bulky monomers to overcome their poor solubility and lowers interfacial tension (Figures S24–S26) [27], thereby promoting amine monomer diffusion into the oil phase and breaking dimensional confinement (Figure 3A and Figures S27 and S28). X‐ray photoelectron spectroscopy (XPS) survey scans reveal elevated O/N ratios, indicating successful formation of low‐crosslinking PA membranes and the presence of low‐resistance transport channels. Owing to its largest molecular volume among the three monomers, the DATP membrane exhibits the lowest crosslinking degree, alongside enhanced hydrophobicity resulting from its higher aromatic ring content (Figure S29). Notably, an O/N ratio approaching 2 suggests a predominantly linear structure, implying the retention of abundant hydrogen‐bond donors and acceptors (Figure 3B, Figures S30 and S31 and Table S1) [6]. Additionally, the narrower bandgap of the terphenyl unit (Figure 3C), relative to phenyl and biphenyl ones, confers higher polarizability. This gain in dispersion interactions strengthens *π‐*‐*π* stacking [28, 29], which compels ordered chain packing.

Grazing‐incidence wide‐angle x‐ray scattering (GI‐WAXS) analysis directly reveals the highly preferential orientation of the DATP membrane (Figure 3D, Figures S32 and S33). The extracted one‐dimensional scattering profiles show characteristic peaks at *Q*_xy_ = 3.59 and 4.44 Å^−1^ and *Q*_z_ = 4.37 Å^−1^. The peak at *Q*_xy_ = 3.59 Å^−1^ corresponds to *π‐*‐*π* stacking, indicating an edge‐on orientation with polymer segments aligned perpendicular to the substrate [30]. The configuration substantially reduces mass transfer resistance (Figure 3E), demonstrating the capability of the OOIP strategy to regulate ordered domains. Specifically, the proper solvent THF stabilizes this metastable arrangement by accelerating the assembly kinetics of the ordered domains. Consequently, the DATP membrane maintained high permeance even when fabricated into a thicker membrane with extended polymerization times (>1 min) (Figures S34 and S35). Furthermore, a sharp scattering peak at about 4.4 Å^−1^, arising from the polymer chains themselves, indicates the presence of interchain hydrogen bonding interactions that stabilize the ordered domains, a conclusion corroborated by well‐defined lattice fringes in high‐resolution transmission electron microscopy (HR‐TEM) images (Figure 3F). Notably, the X‐ray diffraction (XRD) pattern indicates the formation of a crystalline‐amorphous hybrid state (Figure 3G and Figure S36). Combined with the largest interconnected domain area observed in the DATP membrane, we propose that the ordered domains, stabilized synergistically by *π‐*‐*π* stacking and hydrogen bonding interactions, interconnect at their boundaries to form disordered regions, thereby facilitating continuous mass transfer pathways. Collectively, the highly oriented ordered domains reduce intrinsic mass transfer resistance and narrow the membrane pore sizes, whereas the interconnected network of disordered boundaries cooperatively enhances the overall mass transfer efficiency.

### 2.3 Mass Transfer Behavior of Semi‐Crystalline Microporous Polymer Membranes

**FIGURE 4:**
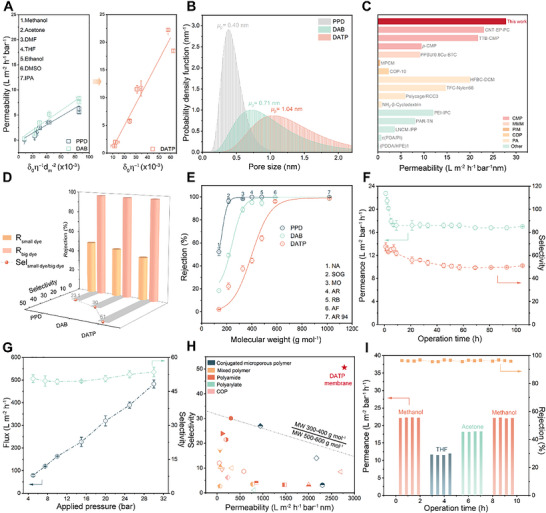
Separation and organic solvent transport behavior of semi‐crystalline microporous polymer membranes. (A) Solvent permeance plots for the three membranes. *η*, solvent viscosity; *δ*_0_, the total solubility parameter; *d*_m_, solvent molar diameter. (B) Probability density function curves derived from the rejection of polyethylene glycol (PEGs) probes of different molecular weights by the three membranes. (C) Comparison of methanol permeability between the DATP membrane and other high‐flux polymer membranes. (D) Selectivity of the three membranes for organic dye mixtures in methanol. (E) Rejection behavior of the three membranes toward selected dyes of different molecular weight in methanol. (F) Long‐term operational stability of the DATP membrane using methyl orange (MO) and acid fuchsin (AF) as markers in methanol. (G) Variation of methanol flux and selectivity of the DATP membrane under varying pressures, using the same markers and solvents as in the long‐term operation. (H) Trade‐off between methanol permeability and selectivity for solutes of different molecular weights (MW), comparing literature‐reported membranes with the membrane fabricated in this work. (I) Alternating flow permeance of methanol, THF and acetone through the DATP membrane, and rejection of AF in these three solvents.

In nanofiltration processes, mass transport through membrane pores is controlled by both solvent‐membrane interactions and steric hindrance [31]. Here, slip length (*L*_s_) is incorporated into the Hagen‐Poiseuille equation to correctly capture flow under confinement [32]:

(1) $$=\frac{\pi \varepsilon r_{p}^{2}}{8\tau d\eta }\times \left(1+\frac{4L_{s}}{r_{p}}\right)=P_{0}\left(1+f_{c}\right)$$

where *P*_0_ is solvent permeance under continuous flow; *η* is solvent viscosity; *d* is membrane thickness, *r*_p_ is pore radius; *ε* is surface porosity; *τ* is membrane tortuosity; *f*_c_ is a comprehensive correction factor accounting for the combined effects of *L*_s_ and *r*_p_ on permeability. We investigated the solvent permeation behavior of the three membranes using seven solvents with distinct properties (Figure 4A). Prior to testing, the membrane was treated with dimethyl sulfoxide (DMSO) to remove oligomers, leveraging the principle of solvent activation (Figures S37 and S38 and Table S2). Hansen solubility parameters (*δ*_0_) were employed to quantify solvent‐membrane interactions (Table S3). Remarkably, the DATP membrane exhibited a substantially enhanced solvent permeability, achieving a methanol permeance of 22.5 L m^−2^ h^−1^ bar^−1^ — a 170% increase over DAB membrane despite its greater thickness. Solvent permeability in the DATP membrane showed a linear relationship with the combination parameter *δ*_0_ *η* ^−1^, indicating that solvent transport is largely unaffected by steric effects, as characterized by solvent molecular diameter. This behavior originates from variations in pore size and the factors that determine the *L*_s_. By combining the rejection data from both dye and PEG probes, we evaluated the effective separation pore size of the DATP membrane and confirmed a larger average pore diameter (*d*_p_ = 1.04 nm), further confirming a positive correlation between *f*_c_ and *δ*_0_ when pore sizes range from 1 to 2 nm (Figure 4B and Figures S39–S41) [31]. This correlation arises from rigidly confined transport channels, where suppressed stacking of polymer chains mitigates the impact of stronger nanoconfinement on mass transport. Such behaviour stands in stark contrast to that observed in the sub‐nanometer pores of the PPD and DAB membranes, where *f*_c_ exhibits a positive correlation with *δ*_0_ *d*_m_^−2^. Meanwhile, this *δ*_0_‐dependent flow entails a violation of the no‐slip boundary condition at the pore wall, where a non‐zero tangential velocity emerges. The consequent increase in *L*_s_ in turn enhances permeability [31]. In addition, the size ratio (*λ*), defined as the quotient of the solvent kinetic diameter (*d*_m_) and the membrane pore diameter (*d*_p_) [33, 34], reveals a critical insight. For the DATP membrane, even the largest solvent molecule (DMF) exhibits a *λ* of less than 0.6, indicating a negligible activation energy (*E*_d_) for diffusion [35]. To quantitatively compare mass transfer efficiency, we calculated the methanol permeability (Figure 4C). With a permeability of 2779 L m^−2^ h^−1^ bar^−1^ nm, the DATP membrane exhibits the highest permeability among polymers of similar molecular weight cut‐off (Table S4). Overall, the synergy of molecular structure and chemical environment maximizes mass transfer efficiency, rendering the DATP membrane exceptionally permeable.

We sought to understand how molecular sieving properties evolve with chain arrangement in semi‐crystalline microporous polymer membranes. Selectivity was assessed using two dyes with molecular weights situated on either side of the molecular weight cut‐off of the membrane [36]. The DATP membrane exhibited a 70% enhancement in selectivity over the amorphous DAB membrane, achieving a selectivity of 51 (Figure 4D,E and Figures S42–S45). Crucially, this selectivity remains constant during long‐term operation (Figure 4F) and under extreme mechanical stress (Figure 4G), encompassing a 110‐hour filtration with a binary feed and applied pressure up to 30 bar. This robustness originates from the high rigidity of the ordered domains, effectively anchoring the transport pathways. A minor decrease in methanol permeance, stabilizing at 17.5 L m^−2^ h^−1^ bar^−1^, is consistent with flow‐induced compaction. Consequently, the DATP membrane delivers the highest selectivity among reported polymer membranes with comparable molecular weight cut‐off (MWCO) (Figure 4H). Given that conventional PA membranes suffer from selectivity loss due to solvent swelling, we further evaluated the chemical stability of the DATP membrane. Static and dynamic tests collectively confirm the chemical stability of the DATP membrane. No changes in membrane morphology or separation performance were observed after 14 days of immersion in various solvents (Figure S46). The DATP membrane withstood 5 days of continuous operation in both polar (THF, DMF, and acetone) and non‑polar (*n*‑hexane) solvents (Figure S47), and showed no performance loss during 10 h of dynamic cycling across three solvents of distinct polarity (Figure 4I). Furthermore, the local structural ordering endows the DATP membrane with outstanding thermal stability, evidenced by an onset decomposition temperature of about 300°C, substantially higher than those of PPD and DAB membranes. This enhancement originates from the significant energy required to disrupt the ordered structures, which leads to a gradual mass loss between 200°C and 500°C due to regional variations in stability (Figure S48).

### 2.4 Separation of Active Pharmaceutical Ingredients

**FIGURE 5:**
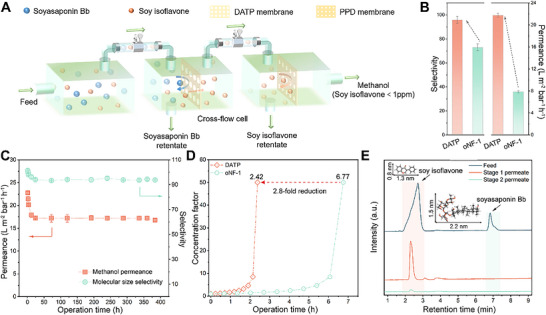
Separation of active pharmaceutical ingredients in methanol. (A) Cascade membrane process for enriching soyasaponin Bb (943.13 g mol^−1^) and recovering methanol from a binary mixture of soy isoflavone (284.26 g mol^−1^) and soyasaponin Bb. (B) Selectivity and methanol permeance of the DATP membrane compared with the commercial BORSIG oNF‐1 membrane. (C) Long‐term permeance and selectivity of the DATP membrane for methanol. The process was conducted in a continuous cross‐flow system under 5 bar transmembrane pressure at a flow rate of 60 L h^−1^. The markers tested were soyasaponin Bb along with soy isoflavone. (D) Concentration factor over time for DATP and commercial BORSIG oNF‐1 membranes. (E) High‐performance liquid chromatography (HPLC) of the feed and permeate at different filtration stages.

Considerable potential exists for employing the semi‐crystalline microporous polymer membrane in the separation of high‐value‐added active pharmaceutical ingredients (APIs). As a demonstrative case, we highlight its efficacy in fractionating soy isoflavone (284.26 g mol^−1^) and soyasaponin Bb (943.13 g mol^−1^) (Figure 5A) [37, 38]. These compounds have rapidly growing markets, driven by their roles in breast cancer prevention and cholesterol reduction, and are projected to reach a combined value of US$1.7 billion by 2026 [39]. The current production of these compounds relies on chromatography, a costly and discontinuous process that limits throughput and efficiency. In contrast, the DATP membrane achieves a high selectivity of 93.8, rejecting >99% of soyasaponin Bb while permitting <20% transmission of soy isoflavone (Figure 5B). Moreover, this high selectivity is maintained over 360 h of continuous operation at a stable permeance of 17.3 L m^−1^ h^−1^ bar^−1^ (Figure 5C). To benchmark this performance, we compared the DATP membrane against a commercial membrane (BORSIG oNF‐1) of a similar molecular weight cut‐off. The DATP membrane achieved a 50‐fold concentration in just 2.5 h, in stark contrast to the 1.1‐fold concentration achieved by the BORSIG oNF‐1 membrane (Figure 5D). Furthermore, the mechanical robustness of the DATP membrane allows for operation at significantly higher pressures, offering a pathway to further reduce energy consumption (Figure S49). Separately, we leveraged the PPD membrane, with its low cross‐linking density imparted by OOIP, for the concentration of soy isoflavone. It exhibited a 4.98‐fold higher permeance compared to a commercial membrane (BORSIG oNF‐2) of similar selectivity, while producing a permeate with a soy isoflavone concentration below 1 ppm, enabling the recovery of high‐purity methanol (Figure 5E). The collective results demonstrate that the semi‐crystalline microporous polymer membrane has the potential to significantly shorten process operation times, while also enabling highly efficient solvent recovery.

## 3 Conclusion

In summary, we proposed a strategy that harnesses intermolecular interactions to constrain segmental motion through ordered stacking and, combined with organic‐organic interfacial polymerization (OOIP), fabricated scalable semi‐crystalline membranes with well‐defined, robust pore architectures. Inspired by articular joints and guided by the marginal contributions of substructures containing aromatic rings identified by a Random Forest (RF) model, we rationally deployed and assembled substructures of distinct functions — using *p*‐terphenyl as the aromatic unit capable of *π*–*π* stacking — to construct ordered domains. These ordered domains are interconnected by disordered regions formed by flexible amide linkages, defining the transport pathways. Implemented by adding THF to the aqueous phase, the OOIP strategy synergistically controls the arrangement of ordered and disordered domains, further lowering mass transport resistance. Specifically, the proper solvent promotes polymer chain assembly kinetics, enabling a metastable edge‐on stacking configuration and breaking dimensionally confined reactions through reduced interfacial tension. The semi‐crystalline membrane (DATP) features low‐resistance, interconnected transport channels with rigid confinement. Compared with amorphous DAB membranes, the DATP membrane shows an approximately 14% increase in FFV (to 35.5%), along with an 14.3% reduction in interconnected volume element loss. Consequently, it achieves a record‐high methanol permeability of 2779 L m^−2^ h^−1^ bar^−1^ nm, combined with the highest selectivity among state‐of‐the‐art polymer membranes with similar molecular weight cut‐off (MWCO), and maintains stable performance for over 110 h. This substantially optimized mass transport kinetics lays the foundation for efficient separation of active pharmaceutical ingredients (APIs). In a separation system for a breast cancer therapeutic agent, the semi‐crystalline polymer membrane achieves a stable selectivity of 93.8, enriches the APIs 50‐fold within 2.5 h, and outperforms commercial membranes by 28% in selectivity. We anticipate that this strategy, endowing polymer membranes with the structural tunability characteristic of crystalline materials, will extend the scope of semi‐crystalline microporous polymer membranes to demanding separations, including hydrocarbon fractionation from crude oil and gas purification, where ultrafast and selective transport is enabled by constrained short‐range ordered domains.

## 4 Experimental Section/Methods

Detailed experimental procedures, synthesis methods, and characterization data are presented in the Supporting Information.

## Author Contributions

**Zi‐Meng Xu**: conceptualization, methodology, data curation, investigation, validation, formal analysis, visualization, writing – original draft. **Hao Zhuo**: methodology, software, validation, formal analysis, writing – original draft. **Song‐Hui Zhi**: investigation, validation, formal analysis. **Xiao‐Feng Zhong**: visualization, investigation. **Pan‐Pan Zhang**: conceptualization, methodology, project administration, formal analysis, writing – review and editing. **Si‐Yuan Yang**: investigation, validation. **Wen‐Yong Yang**: investigation, validation. **Guang‐Yong Shen**: conceptualization, resources. **Ya‐Zhao Li**: conceptualization, resources. **Yi Li**: methodology, data curation, investigation, validation, writing – review and editing. **Ming Xue**: conceptualization, methodology, supervision, project administration, resources, writing – review and editing, funding acquisition, visualization.

## Conflicts of Interest

The authors declare no conflicts of interest.

## Supporting information




**Supporting File**: advs77001‐sup‐0001‐SuppMat.docx.

## Data Availability

The data that supports the findings of this study are available in the Supporting Information of this article.
