# Supplementary material for: Architecture‐Engineered Semi‐Crystalline Microporous Polymer Membranes for Precise Molecular Separation
Source: Adv Sci (Weinh). 2026 Aug 5;13(60):e77001. doi: 10.1002/advs.77001 (PMC13440364; doi:10.1002/advs.77001)
Supplement: Supplementary file 1 [file ADVS-13-e77001-s001.docx]

Supporting Information

**Architecture-Engineered Semi-Crystalline Microporous Polymer Membranes for Precise Molecular Separation**

*Zi-Meng Xu,^1,+^* *Hao Zhuo,^1,+^ Song-Hui Zhi,^1^ Xiao-Feng Zhong,^1^ Pan-Pan Zhang^1,*^Si-Yuan Yang,^1^ Wen-Yong Yang,^1^ Guang-Yong Shen,^2^ Ya-Zhao Li,^2^ Yi Li,^1,*^ Ming Xue,^1,*^*

^[1]^ Z.-M. Xu, H. Zhuo, S.-H. Zhi, X.-F. Zhong, S.-Y. Yang, W.-Y. Yang, Y. Li, M. Xue

School of Chemical Engineering and Technology, School of Chemistry, Southern Marine Science and Engineering Guangdong Laboratory (Zhuhai), OFMMT, Sun Yat-sen University, Guangzhou 510275, P. R. China

E-mail: [xueming5@mail.sysu.edu.cn](mailto:xueming5@mail.sysu.edu.cn); [liyi266@mail.sysu.edu.cn](mailto:liyi266@mail.sysu.edu.cn); zhangpp36@mail.sysu.edu.cn

^[2]^ G.-Y. Shen, Y.-Z. Li
China Lucky Group Corporation, Baoding 071052, P. R. China

^[+]^ Both authors contributed equally to this work.

**The PDF file includes:**

[Experimental Materials and Methods	1](#_Toc213098539)

[Supplementary Figures	9](#_Toc213098540)

[Supplementary Tables	49](#_Toc213098541)

[References	54](#_Toc213098542)

# Experimental Materials and Methods

**Materials**

Polyimide polymer (P84) was purchased from HP Polymer GmbH (Austria) and used for preparing substrate membranes. *p*-Phenylenediamine (PPD, 97%), 4,4*’*-diaminoterphenyl (DATP, 95%), trimesoyl chloride (TMC, 98%), isopropyl alcohol (IPA, 99.9%), *n*-hexane (98%), *N*,*N*-dimethylformamide (DMF, 99.5%), *N*,*N*-dimethylacetamide (DMAc), dimethyl sulfoxide (DMSO) and 1,6-hexanediamine (HDA, 99%) were purchased from Macklin Biochemical Co., Ltd. (Shanghai, China). 4,4*’*-Diaminobiphenyl (DAB, 95%), sudan orange G (SOG, 214.22 g mol^-1^, 95%), methyl orange (MO, 327.3 g mol^-1^, 96.0%), acid red 88 (AR, 452.36 g mol^-1^, 98%), Sudan black B (SBB, 452.36 g mol^-1^, 90%), rhodamine B (RB, 479.0 g mol^-1^, 99%), acid Fuchsin (AF, 585.5 g mol^-1^, 97.0%) and acid red 94 (AR 94, 1017.6 g mol^-1^, 95.0%) were purchased from Aladdin Chemicals Co. Ltd. (Shanghai, China). tetrahydrofuran (THF, 99.0%), methanol (99.5%), ethanol (99.7%), acetone (99.0%), were purchased from Sinopharm Chemical Reagent Co., Ltd. All chemicals were used as received without further purification.

**Methods**

**Preparation of polyimide UF support membranes**

Polyimide (P84) membranes, serving as substrates for thin-film composite (TFC) membranes, were fabricated using a non-solvent-induced phase inversion method. Initially, a homogeneous solution was obtained by dissolving 22 wt% P84 powder in 78 wt% DMF by stirring for 8 hours. The polymer solution, after degassing by standing overnight at room temperature, was carefully cast onto a polyethylene terephthalate (PET) nonwoven fabric fixed to a glass plate with an automatic film casting machine (BEVS 1818G, China) under controlled conditions of 25 ℃ and 70% humidity. To maintain consistent membrane thickness, the gap between the casting knife and the glass plate was set at 250 μm. After casting, the membrane underwent a phase inversion process by immersing it in a 25 ℃ pure water bath for 5 minutes. The resulting P84 ultrafiltration membranes were then placed in a freshwater bath for 2 hours to eliminate residual DMF. Following this, the P84 membranes were crosslinked using HDA to enhance solvent resistance. In summary, after transferring into an IPA bath for 2 hours to eliminate residual water, the P84 membranes were immersed in IPA with 120 g L^−1^ of HDA for 16 hours at room temperature to effect cross-linking. The crosslinked membranes were rinsed with pure water and ultimately stored in pure water at 4 ℃ for subsequent use and characterization.

**Preparation of TFC membranes**

The TFC membranes were prepared via OOIP. Briefly, a P84 ultrafiltration membrane was immersed in an amine monomer solution for 5 minutes, where the concentrations of PPD, DAB, and DATP were 0.2% w/v in a mixed solvent of THF and water (THF:H_2_O = 1:1 v/v). Excess solution was then removed with an air knife, followed by the addition of an *n*-hexane solution containing TMC (0.1% w/v) to the membrane surface for 1 minute to form the polyamide layer. Subsequently, the newly formed composite membrane was rinsed with *n*-hexane and dried in an oven at 60 ℃ for 10 minutes. Finally, the composite membrane was rinsed with and stored in pure water until use.

**Solvent activation of TFC membranes**

The influence of solvent activation on membranes was investigated. The TFC membranes were immersed in an activation solvent, DMSO under static conditions at room temperature. After treatment for a predetermined period, the membrane was thoroughly rinsed with pure MeOH to eliminate any remaining DMSO and then stored in pure MeOH for subsequent separation tests.

**Synthesis of the freestanding nanofilm and polymer powder**

Using the same parameters as for the fabrication of TFC membranes, freestanding nanofilms and polymer powders were synthesized. The freestanding nanofilms were formed at the organic-organic interface by slowly adding a solution of TMC in *n*‑hexane to an amine‑containing organic solution. After one minute, the silicon wafer supporting the polyamide membrane was rinsed with *n*‑hexane for further characterization. Polymer powders were prepared by vigorously mixing the amine-containing organic solution with the TMC-containing organic solution, collecting the resulting polymer films, and thoroughly drying them in a vacuum oven at 120 °C for 12 hours. The dried polymer powders were then stored in a desiccator for further characterization.

**Characterizations**

The chemical compositions of the PA layer were analyzed using X-ray photoelectron spectroscopy (XPS, Nessa, Thermos Fisher Scientific, USA) with a monochromatic Al Kα X-ray source. The parameters were set to a total acquisition time of 68s in CAE Analyzer Mode with a passing energy of 150.0 eV. The spot size was 400 mm, and the vacuum level in the analysis room was approximately 10^-5^ mbar. The C1s signal of aromatic carbon 284.5 eV was used as a reference to calibrate binding energy data. The charge properties of the membrane surface were measured by a Zeta potential analyzer (Surpass 3, Anton Paar, Austria) with a KCl electrolyte solution of 0.001 mol L^−1^. The static contact angle was tested using a Data-Physics OCA20 with a 2 ml water droplet. The morphology of the PA layers was observed by high-resolution cold-field emission scanning electron microscope (SEM, Regulus-8230, Hitachi, Japan), and atomic force microscope (AFM, Dimension icon, Bruker, USA). The cross-sectional structure was examined by transmission electron microscopy (TEM, JEM-F200, JEOL, Japan) at an accelerating voltage of 100 kV. Attenuated Total Reflection-Fourier transform infrared (ATR-FTIR, Nicolet Is50, Thermo FiSher, USA) spectra was obtained on a Nicolet iS10 spectrometer. Thirty-two scans and a resolution of 4 cm^−1^ were set. Thermogravimetric analysis (TGA, TG 209F1 Libra, NETZSCH, Germany) was implemented on the TA-Q500 with a temperature ramp of 10 °C min^−1^ and a nitrogen flow rate of 10 mL min^−1^. The Brunauer-Emmett-Teller (BET) technique was used to determine the texture of the microporosity in the polymer powder using a Micromeritics ASAP™ 2020 system (Atlanta, GA, USA) with micropore option. Powder polymer samples (50 mg) were degassed under high vacuum at 120°C for 12 hours prior to analysis. All data were collected and analyzed using ASAP-2020 software version 4.02. CO_2_ gas was used for sorption tests at 273 K and absolute pressures of 0-1 bar. Non-localized density functional theory (NLDFT) was used to obtain qualitative pore size distributions assuming carbon slit-like pores.

**Applications of Machine Learning Models**

The dataset used in this work was sourced from the first large-scale polymer FFV database compiled by Li et al.,^[1]^ which comprises 8046 SMILES strings representing the repeating units of homopolymers, along with their corresponding fractional free volume (FFV) values.^[2]^ All 8046 SMILES strings were successfully parsed and validated as molecular structures using RDKit.^[3]^ Among these, 7892 unique structures were identified, with 154 duplicate SMILES entries. The molecular weight of the polymers in the dataset ranges from 14 to 2203 g mol^-1^ (mean: 459 g mol^-1^), and the number of aromatic rings per structure ranges from 0 to 15 (mean: 3.29). Polymers containing at least one aromatic ring account for 84.6% of the dataset (6807 entries). The FFV target values exhibit a distribution ranging from 0.227 to 0.777, with a mean of 0.367, a median of 0.364, and a standard deviation of 0.029. The distribution is positively skewed (skewness: 1.99) and leptokurtic (kurtosis: 19.24), indicating a highly concentrated data population with a long tail extending towards high FFV values. Specifically, 88.4% of the samples fall between 0.31 and 0.40, 8.0% fall between 0.40 and 0.43, and 2.3% fall between 0.43 and 0.50. Only 15 samples (0.2%) exhibit FFV values exceeding 0.50, while merely 12 samples (0.1%) fall below 0.28.

Feature engineering workflow was performed as follows. First, Morgan fingerprints were computed for the repeating unit of each polymer from its SMILES string using RDKit,^[4]^ with a radius parameter of 3 to capture local substructural information within a radius of three bonds from each atom. This procedure yielded 34876 unique Morgan bits from the 8046 polymers, each corresponding to a specific local substructure pattern and valued by its occurrence count in the respective polymer. To eliminate sparse features, we applied a zero-count threshold: bits with zero values in more than 7900 polymers (i.e., appearing in fewer than 146 polymers) were discarded. This filtering retained 425 features. Subsequently, based on RDKit ring information, we determined whether the subgraph covered by each Morgan bit contained at least one complete aromatic ring, defined as a ring satisfying the aromaticity criterion. Among the 425 retained features, 86 were identified as aromatic-ring-related substructures, accounting for 20.2% of the total.

The dataset was randomly split into training and test sets at an 80: 20 ratio with a fixed random seed of 26 to ensure reproducibility, resulting in 6436 samples for training and 1610 for testing (test_size_ = 0.20, random_state_ = 26 in the train_testsplit_ function). For model development, we constructed two machine learning models, a Random Forest (RF) regressor and an XGBoost gradient-boosting regressor, using identical training-test splits to enable direct comparison. The RF model was configured with 100 trees, a maximum depth of 30, and with bootstrap sampling disabled so that each tree was trained on the full training set. The feature subsampling strategy was set to the square root of the total number of features, selecting approximately 20 features at each split. The XGBoost model was configured with 180 boosting rounds, a maximum tree depth of 14, a learning rate of 0.08, a row sampling rate of 0.81, a column sampling rate of 0.80, and a gamma regularization parameter of 0. All models were initialized with random_state_ = 26 to ensure reproducibility. Model performance was evaluated using four metrics: R^2^, MAE, RMSE and MSE. On the test set, the RF model achieved an R^2^ of 0.868, an MAE of 0.0064 and an RMSE of 0.0102, while the XGBoost model achieved an R^2^ of 0.876, an MAE of 0.0057 and an RMSE of 0.0099. The two models exhibited comparable performance. For model interpretability, SHapley Additive exPlanations (SHAP) analysis was performed using the TreeExplainer method to compute SHAP values for each feature.^[5]^ This allowed us to identify the top 20 most important Morgan substructure features globally, as well as the top 20 most important aromatic-ring-related features, for which corresponding substructure visualizations were generated.

**Molecular simulation**

Molecular dynamic (MD) methods were employed to simulation three considered membrane structures, followed by pore landscape distribution analysis by Zeo++ code. The three systems each consisting of 15 structure segments were randomly packed into cubic simulation boxes. Then, each model was subjected to structure optimization, annealing and equilibrium MD simulation. All MD simulations were carried out by Forcite module with COMPASS III force field in Materials Studio (MS) 2020. ^[6,7]^ Van der Waals and Coulomb interactions were respectively considered by atom based and Ewald methods with a cut-off value of 12.5 Å. Equations of motion were integrated with a time step of 1 fs. After energy minimization, each system was subjected to 20 circle annealing simulation with temperature from 298 K to 500 K. Then the lowest energy configurations were fully relaxed under periodic boundary conditions for 800 ps in the NPT (*P* = 1 atmosphere, *T* = 298.0 K) ensemble using the Nose thermostat and Berendsen barostat, which was long enough for system temperature, potential and total energy to get stable. The final equilibrium state configuration was analyzed by Zeo++ (version 0.3) and atom volumes & surfaces tool in MS.^[8-10]^

**Calculation of crosslinking degree of polyamides**

The degree of network crosslinking (m) of the polyamide membrane was determined by the atomic ratio of nitrogen to oxygen (N/O) obtained from XPS elemental analysis. Its relationship with the degree of crosslinking (*n*) in linear polyamide membranes is described by the following equation:^[11]^

$$\begin{aligned} m+n=1\#\left( S1 \right) \end{aligned}$$

Here, a fully crosslinked network denotes a structure in which every acyl chloride group from TMC and every amine group from the diamine monomer are utilized to form amide linkages. Consequently, each TMC molecule connects to 1.5 diamine monomers. In contrast, a perfectly linear structure implies that only two of the three acyl chloride groups in TMC participate in polyamide formation, leaving one acyl chloride group unreacted, which ultimately hydrolyzes to form a carboxylic acid.

For PPD, DAB, and DATP membrane:

$$\begin{aligned} \frac{N}{O}=\frac{3m+2n}{3m+4n}\#\left( S2 \right) \end{aligned}$$

We propose that the extent of reaction for the amine moiety can be derived from the deconvolution of high-resolution N 1s XPS spectra, as indicated by the relative proportion of amide bonds.

**Measurement of membrane pore size**

Considering that the MWCO does not accurately reflect the relationship between membrane pore size and molecular diameter, the geometric mean diameter of the molecule (*D*_geo_) was selected as the measure of molecular size. The lowest-energy conformation of each molecule was generated using Chem3D,^[12]^ from which the spatial coordinates of all constituent atoms were obtained. On this basis, and using the three orthogonal molecular dimensions together with the van der Waals (vdW) radii of the atoms, the geometric mean diameter (*D*_geo_) was calculated. *D*_geo_ was determined according to the following equation:^[13]^

$$\begin{aligned} D_{\mathrm{geo}}=\sqrt[3]{d_{1}\times d_{2}\times d_{3}}\#\left( S3 \right) \end{aligned}$$

where *d*_1_, *d*_2_ and *d*_3_ represent the molecular dimensions along three orthogonal axes. The geometric mean diameter is less sensitive to extreme dimensional values than the arithmetic mean and better reflects the overall molecular volume.

By plotting molecular diameter as a function of rejection, we confirmed that molecules with different charges exhibited a strong linear relationship between molecular diameter and rejection. This linear relationship was then used to estimate the molecular diameter corresponding to 90% rejection.^[14]^

Ferry’s equation was used to estimate the membrane pore size, which was calculated as follows:^[15]^

$$\begin{aligned} R=1-{2\left( 1-\frac{D}{P} \right)}^{2}+\left( 1-\frac{D}{P} \right)^{4}\#\left( S4 \right) \end{aligned}$$

where *R* is the rejection, *D* is the diameter of the molecules, and *P* is the membrane pore size.

We further determined the pore size of the polyamide membrane by measuring the rejection of a series of neutral polyethylene glycol (PEG) probes with increasing molecular weights (62, 200, 400, 600, 800 and 1000 Da). The concentration of each solute species solution was 100 ppm and the applied pressure in the filtration experiments was 5 bar. PEG concentration in the feed and permeate were determined using TOC-VCPH analyze. The MWCO of TFC- membranes was defined as the molecular weight at which the rejection equals 90%. The pore size distribution curve is expressed as a probability density function (PDF) that was established based on the following assumption: (1) There is no hydrodynamic and electrostatic interactions between these neutral solutes and the membrane pores; (2) The geometric mean diameter (*μ*_p_) of the membrane equals the Stokes diameter of a solute when the rejection is 50%; (3) The distribution of the membrane pore size is characterized by the geometric standard deviation (*σ*_p_) of the PDF curve, which is the ratio between the Stokes diameter with a rejection of 84.13% to that with a rejection of 50%.^[16]^

$\begin{aligned} \frac{dR\left( d_{p} \right)}{dd_{p}}=\frac{1}{d_{p}\ln\sigma_{p}\sqrt{2\pi}}exp\left[ -\frac{\left( \ln d_{p}-\ln\mu_{p} \right)^{2}}{2\left( \ln\sigma_{p} \right)^{2}} \right]\#\left( S5 \right) \end{aligned}$where *m*_p_ is the geometric mean diameter of the membrane, *s*_p_ is the geometric standard deviation of the PDF curve and *d*_p_ is the pore size of the membrane. The Stokes diameter of PEG molecules correlate with their molecular weight:

$$\begin{aligned} a=16.73\times{10}^{-10}\times M_{PEG}^{0.557}\#\left( S6 \right) \end{aligned}$$

$$\begin{aligned} d_{s}=2\times a\times{10}^{7}\#\left( S7 \right) \end{aligned}$$

Where a is the Stokes radius (nm), *M*_PEG_ is the molecular weight of PEG.

Dye rejection measurements give a DATP membrane pore size of 1.01 nm, which closely matches the value obtained from PEG rejection (1.04 nm). This consistency confirms that the separation performance of the DATP membrane is governed primarily by physical size exclusion.

**Membrane performance evaluation**

Pure solvent filtration and OSN tests were conducted using a standard dead-end cell (Sterlitech, HP4750). The cell was pressurized using an external N_2_ cylinder. The effective test area inside the cell was 14.6 cm^2^, and the test pressure was 5 bar. Magnetic stirring (500 rpm) was employed to eliminate concentration polarization when testing dye solutions. For each test, the membrane was compacted at 5 bar for at least 30 min until stable, and the permeate was collected for 15 min. The permeance (L m^-2^ h^-1^ bar^-1^) of the TFC membrane was calculated using the following formula:

$$\begin{aligned} P=\frac{V}{A\times t\times\Delta P}\#\left( S8 \right) \end{aligned}$$

where *V* (L) was the permeate volume, *A* (m^2^) was the effective filtration area, *t* (h) was the collection time, and$\Delta P$ (bar) was the testing pressure.

The following formula (S9) was used to calculate the membrane rejection (%):

$$\begin{aligned} R=\left( 1-\frac{C_{p}}{C_{f}} \right)\#\left( S9 \right) \end{aligned}$$

where $C_{p}$ and $C_{f}$were the solute concentrations of the permeate and feed solution, as determined by UV-vis spectrophotometry. The feed concentration for the dye separation test was 50 ppm. All data reported were obtained from at least three parallel samples and are reported as the average value of multiple tests.

Selectivity was calculated as the concentration ratio of the larger and smaller molecules. A binary system of soyasaponin Bb and soy isoflavone was selected because the membrane pore size falls between their molecular dimensions. Both drugs were at a concentration of 50 ppm. The separation performance of the three membranes was evaluated using a cross-flow filtration system at 30 °C, with an effective membrane area of 32 cm^2^. Selectivity is given by the following equation:

$$\begin{aligned} Selectivity=\frac{C_{permeate,small}/C_{feed,small}}{C_{permeate,large}{/C}_{feed,large}}\#\left( S10 \right) \end{aligned}$$

where *C*_feed, large_ and *C*_permeate, large_ denote the concentration of soyasaponin Bb (the larger molecule), in the feed and permeate, respectively; *C*_feed, small_ and *C*_permeate, small_ represent the concentration of soy isoflavone (the smaller molecule) in the feed and permeate, respectively.

This experiment was conducted using a Waters Arc HPLC system equipped with a 2489 UV/Vis Detector. The detection wavelength was set at 205 nm. Chromatographic separation was performed on an Agilent ZORBAX SB C18 column (4.6 × 250 mm) at 40 °C. The mobile phase consisted of methanol and water (70:30, v/v) at a flow rate of 1.0 mL min^-1^ under isocratic elution for 10 minutes. The injection volume was 10 μL. For sample preparation, 0.5 mL of the sample was diluted to 1.0 mL with 75% methanol in water. After thorough mixing, 1 mL of the resulting solution was subjected to HPLC analysis.

**Analysis of energy consumption**

The energy consumption (kWh) during membrane operation was calculated from the power (kW) of the pump and chiller multiplied by their operating time (h). The pump was operated at its maximum frequency of 50 Hz, corresponding to a rotational speed of 1400 rpm and a flow rate of 3.0 gpm.^[14]^ The pump power (*P*_pump_) was determined using the following equation:

$$\begin{aligned} P_{\mathrm{pump}}=\frac{6\times1400}{63000}+\frac{3\times AP}{1460}\#\left( S11 \right) \end{aligned}$$

Here, *AP* denotes the applied pressure (psi). The chiller power remained constant at 0.2 kW. For example, at an applied pressure of 5 bar (72.5 psi), *P*_pump_ was determined to be 0.16 kW, resulting in a combined power consumption of 0.36 kW for the pump and chiller. Operating for 1 hour under these conditions would therefore yield an energy consumption of 0.36 kWh.

The energy consumption for distillation (bench-scale) was calculated based on the power inputs of the vacuum rotary evaporator and the cooling circulation pump. The water bath temperature was maintained at 35 °C throughout the experiment, resulting in a constant heating power of 0.348 kW. The combined power for the remaining components was 1.355 kW. The total energy consumption for distillation, was therefore calculated using the following equation:

$$\begin{aligned} E_{\mathrm{distillation}}=1.355t+0.348\#\left( S12 \right) \end{aligned}$$

Here, *t* denotes the operation time (h).

The enrichment time was estimated as the duration required to concentrate a 5 L methanol solution of soyasaponin Bb (943.13 g mol^-1^) from an initial 50 mg L^-1^ to a final concentration of 2.5 g L^-1^. The effective filtration area of the membrane is 192 cm^2^. The process was conducted in a continuous cross-flow system under 5 bar transmembrane pressure at a flow rate of 60 L h^-1^.

# Supplementary Figures

**Figure S1.** Schematic representation of the reaction mechanism and possible chemical structures for polyamide formation from TMC and three amine monomers (PPD, DAB and DATP, respectively).

**Figure S2.** FT-IR spectra of the PPD, DAB and DATP membranes.

**Note:** The three membranes exhibited four characteristic peaks, which were assigned to amide C=O (1630-1650 cm^-1^), amide N-H (1490-1510 cm^-1^), imide C-N (1400 cm^-1^), and imide ring (1180 cm^-1^).

**Figure S3.** Comparison of solid-state ^13^C CP-MAS NMR spectra of DATP monomer and DATP membrane.

**Figure S4.** Scale-up preparation of substrate by non-solvent induced phase separation (NIPS) and the DATP membrane by interfacial polymerization (IP).

**Figure S5.** (A) Photographs and surface SEM images of the large-area DATP membrane cut from the front, middle, and rear sections. (B) OSN performance of nine samples collected from the front, middle, and rear sections of the large-area DATP membrane, using AF as the marker dye in methanol.

**Figure S6.** (A) Digital photographs, (B) surface SEM images, and (C) OSN performance of the DATP membranes prepared from different batches, using AF as the marker dye in methanol.

**Figure S7.** Photographs of the (A) PPD, (B) DAB, and (C) DATP membranes (diameter = 5 cm).

**Figure S8.** AFM surface morphology of the (A) PPD, (B) DAB and (C) DATP membranes.

**Figure S9.** Cross-sectional TEM images of the (A) PPD, (B) DAB and (C) DATP membranes.

**Figure S10.** SEM surface morphology of the (A) PPD, (B) DAB and (C) DATP membranes.

**Figure S11.** Simulated energy-minimized structures and their cross-sections for amorphous cells of the (A) PPD, (B) DAB, and (C) DATP membranes. Grey: polymer chains; green: accessible free volume at a probe radius of 1 Å; and black: periodic cell with dimensions of 39.5 × 39.5 × 39.5 Å^3^. The cross-sections are 10 Å in thickness.

th

**Figure S12.** Simulated free volumes and densities of the three membranes.

**Figure S13.** Simulated pore size distribution of the PPD, DAB and DATP membranes.

**Figure S14.** Differences in the filled volume faction of the DAB and DATP membranes after aging tests.

**Note:** Following aging tests, methanol molecules permeate the membrane. Under steady-state operation, the semi-crystalline DATP retains a greater number of interconnected pores compared to the DAB membrane, which facilitates enhanced mass transport within the membrane.

**Figure S15.** Pore size distribution for the PPD, DAB and DATP membranes derived from CO_2_ sorption isotherms based on NLDFT calculations.

**Note:** Given that polymer membranes exhibit differences in apparent pore size between dry and wet states, the pore size distribution reported here pertains solely to that under dry conditions.

**Figure S16.** Young’s modulus of the (A) PPD, (B) DAB and (C) DATP membranes tested by peak force quantitative nanomechanical mapping (PFQNM) methods.

**Figure S17.** (A) Substructure scanning across varying radii. (B) Substructural feature matching and visualization.

**Note:** Complete procedure for substructure identification and extraction via molecular fragment fingerprint (MFF.)(**1)** **Molecular-structure representation:** The molecular structure of PIM-1 is converted into a graph representation, where each atom is a node and each chemical bond is an edge connecting two nodes. Node attributes encode atomic information (e.g., C, O, or N), whereas edge attributes encode bond information (e.g., single, double, or aromatic bonds).

(**2)** **Definition of the scanning radius:** A scanning radius for the MFF, denoted as *r*, is then specified. *r* defines the maximum topological distance over which the local environment is expanded from a given central atom along chemical bonds. For example, when *r* = 1, the fingerprint captures the central atom and its directly bonded neighbours. When *r* = 2, it includes the central atom, its first-neighbour atoms, and the next shell of connected atoms. When *r* = 3, all atoms within three bond distances from the central atom are retained. Thus, increasing *r* enables the extracted substructure to encode a richer local chemical environment.

**(3) Selection of the central atom:** Each atom in the molecule is sequentially selected as the central atom for MFF scanning. In PIM-1, such atoms may include C atoms in aromatic rings, O atoms in ether linkages, C or N atoms in nitrile groups, and C atoms near the spiro-centre. Therefore, every atom in the molecular graph can serve as the starting point for substructure extraction.

**(4) Expansion within the scanning radius:** Starting from the central atom, the molecular graph is traversed along chemical bonds. For a given radius *r*, all atoms and bonds located within a topological distance of *r* from the central atom are retained. For example, if an ether oxygen atom is selected as the central atom, the substructure at *r* = 0 contains only the O atom. At *r* = 1, it contains the O atom and the two directly bonded C atoms, corresponding to a C-O-C fragment. At *r* = 2, the substructure further includes atoms connected to these C atoms, such as adjacent aromatic carbon atoms. At *r* = 3, the extracted fragment extends to a larger local segment of the aromatic ring.

**(5) Recording of local substructures:** After expansion, the resulting set of local atoms and bonds is recorded as a substructure. This substructure contains information on atom types, atomic connectivity, bond types, aromaticity, ring motifs and specific functional groups, such as ether linkages, nitrile groups and aromatic rings. For PIM-1, the captured substructures may include aromatic-ring fragments, C-O-C ether linkages, -C≡N nitrile fragments, rigid segments near the spiro-centre and oxygen-containing heterocyclic fragments.

**(6) Encoding of substructures:** Each captured substructure is then converted into a unique code or fingerprint bit. Identical local structures are mapped to the same feature index, whereas distinct local structures are assigned different indices. For instance, a specific C-O-C local environment corresponds to one fingerprint feature, an aromatic C-C-C fragment to another, and a -C≡N fragment to a separate feature. Collectively, all extracted and encoded substructures constitute the MFF molecular fingerprint of the polymer.

**Figure S18.** Predictive performance of the (A) RF and (B) XGBoost models.

**Figure S19.** (A) Global interpretation of the RF model via SHAP and (B) twenty most critical substructures contributing to the model prediction.

**Figure S20.** (A) Key substructures identified by the RF model and (B) twenty most critical aromatic-ring-containing substructures contributing to the model prediction.

**Figure S21.** (A) Global interpretation of the XGBoost model via SHAP and (B) twenty most critical substructures contributing to the model prediction.

**Figure S22.** (A) Key substructures identified by the XGBoost model and (B) twenty most critical aromatic-ring-containing substructures contributing to the model prediction.

**Figure S23.** Comparison of the conventional IP reaction and organic-organic IP reaction.

**Note:** THF was added to the aqueous phase to dissolve the water-insoluble amine monomer. In addition, the amine-dominated diffusion reaction results in the formation of a relatively rough and loose polyamide layer, which is attributed to the reduced interfacial tension, allowing for less cross-linking of the polyamide.

**Figure S24.** Time course of TMC concentration changes in the solvent-water phase.

**Figure S25.** The surface tension of solvent-water and the interfacial tension between solvent-water and *n*-hexane.

**Figure S26.** (A) Schematic representation of the Connolly surface area of the three monomers. (B) Length, (C) surface area, and (D) occupied volume by the monomer in its energy-minimized state.

**Figure S27.** Optical photographs of the free-standing PPD, DAB and DATP nanofilm formed at the THF-H_2_O/*n*-hexane interface.

**Figure S28.** Schematic diagram of the measurement of (A) the height of UV-light, (B) test point in aqueous solution, and (C) test point in organic solution.

**Figure S29.** Water contact angles of the PPD, DAB and DATP membranes.

**Figure S30.** High-resolution O1s XPS spectra of the (A) PPD, (B) DAB and (C) DATP membranes. (D) Variation in O=C-O* bonding ratio.

**Figure S31.** High-resolution C1s and N1s XPS spectra of the (A, D) PPD, (B, E) DAB and (C, F) DATP membranes.

**Figure S32.** 2D GI-WAXS of the DAB membrane.

**Figure S33.** Schematic of possible molecular orientations in nanofilms.

**Note:** Crystallites with no preferred orientation relative to the substrate normal produce ring-shaped diffraction patterns, whereas films with orientationally distributed grains show arced patterns. Highly oriented films, in contrast, yield spot-like diffraction patterns.

**Figure S34.** SEM morphology of composite and free-standing membranes prepared by different reaction times.

**Figure S35.** Methanol permeances of the fabricated (A) DAB and (b) DATP membranes with different reaction times.

**Figure S36.** XRD patterns of the three membranes.

**Figure S37.** Dye rejection in methanol of the (A, B) PPD, (C, D) DAB and (E, F) DATP membranes before and after solvent activation.

**Figure S38.** SEM surface images of the DATP membranes (A, C) before and (B, D) after activation.

**Figure S39.** Linear correlation between Stokes diameter and rejection for the (A) PPD, (B) DAB and (C) DATP membranes. (D) PEG rejection curves of the PPD, DAB and DATP membranes.

**Figure S40.** Surface zeta potential curves of the PPD, DAB and DATP membrane vary with pH.

**Figure S41.** Linear correlation between geometric mean diameter of dyes and rejection for the PPD, DAB and DATP membranes.

**Figure S42.** Photographs of the DATP membrane (A) before and (B) after dye rejection tests using positive (left) and negative (right) charged dyes, respectively.

**Figure S43.** UV-vis spectra of feed and permeate of molecules before and after filtration by the DATP membrane.

**Figure S44.** (A) UV/Vis absorption spectra and (B) schematic diagram of a mixture of MO and AF in methanol before and after filtration through the DATP membrane.

**Figure S45.** (A) UV-vis absorption spectra of the feed solution and the permeate: MO, AF, methyl blue (MB) and Congo red (CR). (B) Schematic of the dye separation performance of the DATP membrane.

**Figure S46.** (A) Photographs of free-standing DATP nanofilms in several organic solvents. (B) Filtration performance of the DATP membrane after methanol immersion treatment.

**Figure S47.** Long-term permeance of the DATP membrane in (A) *n*-hexane, (B) THF, (C) DMF and (D) acetone.

**Figure S48.** TGA curves of the PPD, DAB and DATP membranes under N_2_ flow.

**Figure S49.** Energy consumption variations under different operating pressures.

# Supplementary Tables

**Table S1.** Elemental composition and O/N ratio of the PPD, DAB and DATP membranes.

| Membranes | Concentration (at. %) | | | Ratio (%) | | Degree of reaction (%, amine) |
| --- | --- | --- | --- | --- | --- | --- |
|  | C 1s | N 1s | O 1s | | O/N |  |
| PPD | 73.06 | 10.76 | 16.18 | | 1.50 | 82.8 |
| DAB | 77.79 | 7.65 | 14.56 | | 1.90 | 88.1 |
| DATP | 79.93 | 6.41 | 13.66 | | 2.13 | 80.1 |

**Table S2.** Dye rejection in methanol by the PPD, DAB and DATP membranes at different activation times. The MWCO for DATP membranes remains the same before and after activation.

| Membranes | Pure methanol  (L m^-2^ h^-1^ bar^-1^) | Dye rejection (%) | | | |
| --- | --- | --- | --- | --- | --- |
|  |  | SOG | MO | AF | RB |
| PPD | 2.0±0.1 | 96.4±2.0 | 98.3±0.3 | 99.4±0.1 | 99.7±0.1 |
| PPD-30 | 6.2±0.5 | 69.3±1.5 | 97.6±1.5 | 99.7±0.1 | 98.5±0.7 |
| PPD-60 | 7.7±0.3 | 58.3±3.0 | 95.1±3.0 | 99.6±0.1 | 99.7±0.1 |
| DAB | 2.3±0.2 | 84.3±0.7 | 95.3±1.2 | 99.8±0.1 | 99.9±0.1 |
| DAB-30 | 8.3±0.2 | 49.2±1.4 | 90.0±2.2 | 99.7±0.2 | 98.7±1.7 |
| DAB-60 | 11.8±0.4 | 38.8±1.4 | 82.2±2.7 | 99.5±0.1 | 98.6±1.7 |
| DATP | 3.2±0.2 | 24.2±2.4 | 35.5±2.5 | 96.8±0.3 | 98.5±0.4 |
| DATP-30 | 21.2±1.3 | 20.1±3.6 | 39.3±1.7 | 95.4±0.7 | 99.0±0.7 |
| DATP-60 | 21.5±2.7 | 21.6±1.1 | 38.7±3.6 | 95.7±3.1 | 98.8±1.0 |

**Table S3.** Hansen solubility parameter and other physical properties of organic solvents used in this work.

| Solvent | Viscosity (mPa s) ^a^ | Hansen solubility parameter (MPa^1/2^) ^b^ | | | |
| --- | --- | --- | --- | --- | --- |
|  |  | *δ*_d_ | *δ*_p_ | *δ*_h_ | *δ*_0_ |
| Dimethylformamide | 0.816 | 17.4 | 13.7 | 11.3 | 24.8 |
| Ethanol | 1.074 | 15.8 | 8.8 | 19.4 | 26.5 |
| Methanol | 0.6 | 15.1 | 12.3 | 22.3 | 29.7 |
| Isopropanol | 2.04 | 15.8 | 6.1 | 16.4 | 23.5 |
| Dimethyl sulfoxide | 1.996 | 18.4 | 16.4 | 10.2 | 26.7 |
| Acetone | 0.306 | 15.5 | 10.4 | 7.0 | 20.1 |
| Tetrahydrofuran | 0.55 | 16.8 | 5.7 | 8.0 | 19.4 |

^a^) Viscosity is at 25℃ and is taken from ref; ^[17]^ ^b^) *δ*_d_ = Solubility parameter due to dispersion forces, *δ*_p_ = solubility parameter due to dipole forces, and *δ*_h_ = solubility parameter due to hydrogen bonding (or in general due to donor-acceptor interactions).

**Table S4.** Comparison of state-of-the-art polymer-based OSN membranes reported in the literature.

| Membrane | MWCO  (g mol^-1^) | Permeance  (L m^-2^ h^-1^ bar^-1^) | Thickness  (nm) | Permeability (L m^-2^ h^-1^ bar^-1^ nm) | Selectivity | Ref. |
| --- | --- | --- | --- | --- | --- | --- |
| γ-CDA-TPC-0.05 | 450 | 9.87 | 20 | 197.4 | 21.5 | ^[18]^ |
| SC [4] AA -TPC-0.1 | 450 | 3.08 | 20 | 61.6 | 2.7 | ^[18]^ |
| *β*-CD-1.0 | 520 | 5.4 | 500 | 2700 | 8.5 | ^[19]^ |
| *β*-CD-NH_2_ | 450 | 17 | 6.3 | 107.1 | 9.5 | ^[20]^ |
| MPCM | 450 | 14 | 3.5 | 49 | 12 | ^[21]^ |
| c(PDA/PI) | 460 | 1.8 | 40 | 72 | 10.5 | ^[22]^ |
| *p*-CMP | 520 | 22.5 | 42 | 945 | 27 | ^[23]^ |
| *m*-CMP | 530 | 16.5 | 45 | 742.5 | 24.3 | ^[23]^ |
| *p*-CMP-OOC11 | 550 | 1.36 | 40 | 54.4 | 5.3 | ^[23]^ |
| APIMO/TMC | 600 | 31 | 25 | 775 | 3.5 | ^[24]^ |
| F_5_-TAB | 560 | 3.5 | 92 | 322 | 10.8 | ^[14]^ |
| COP-10 | 500 | 16.5 | 14 | 231 | 6.2 | ^[25]^ |
| COP-30 | 500 | 11 | 30 | 330 | 2.7 | ^[25]^ |
| COP-60 | 480 | 7.5 | 32 | 240 | 2.2 | ^[25]^ |
| HFBC-DCM | 600 | 40 | 50 | 2000 | 3.2 | ^[26]^ |
| PI/TA-MoS_2_ TFN | 460 | 21 | 15 | 315 | 10 | ^[27]^ |
| LNCM /PP | 582 | 7.5 | 48.6 | 364.5 | 8 | ^[28]^ |
| TTB-CMP | 495 | 31 | 70 | 2170 | 14 | ^[29]^ |
| TFC-Nylon66 | 550 | 35 | 42 | 1470 | 3.25 | ^[30]^ |
| F_5_N_6_F_5_ | 450 | 19 | 12.3 | 233.7 | 21 | ^[31]^ |
| F_9_N_6_F_9_ | 450 | 16.5 | 18.5 | 305.3 | 3.3 | ^[31]^ |
| CNT-EP-PC | 540 | 23 | 100 | 2300 | 7.5 | ^[32]^ |
| DATP | 560 | 22.5 | 123.5 | 2778.75 | 50.6 | This work |

**Table S5.** Chemical structures and properties of the dyes used for the separation experiments.

| Dye name | M_w_  （g mol^-1^） | Structure | Diameter (nm) | Charge |
| --- | --- | --- | --- | --- |
| *p*-Nitroaniline (NA) | 138.1 |  | 0.57 | 0 |
| Sudan Orange G (SOG) | 214.2 |  | 0.71 | 0 |
| Methyl orange  (MO) | 327.3 |  | 0.86 | -1 |
| Acid Red 88  (AR) | 400.8 |  | 1.01 | -1 |
| Rhodamine B  (RB) | 479.0 |  | 1.25 | +1 |
| Acid Fuchsin (AF) | 585.5 |  | 1.15 | -2 |
| Acid Red 94  (AR 94) | 1017.6 |  | 1.28 | -2 |

# References

[1]	L. Tao, J. He, T. Arbaugh, et al., "Machine learning prediction on the fractional free volume of polymer membranes," *J. Membr. Sci.* (2023): *665*, 121131.

[2]	S. Guo, C. Fang, J. Li, et al., "Microporous Polymer Membranes: Molecular Stents Enhanced Solvent-Accessibility for Organic Solvent Transport," *Adv. Sci.* (2025): *12*, e16748.

[3]	G. Landrum, "RDKit: A software suite for cheminformatics, computational chemistry, and predictive modeling.," *Greg Landrum* (2022.9.5).

[4]	J. Yang, L. Tao, J. He, et al., "Machine learning enables interpretable discovery of innovative polymers for gas separation membranes," *Sci. Adv.* *8*, eabn9545.

[5]	M. Wang, J. Jiang, "Accelerating Discovery of High Fractional Free Volume Polymers from a Data-Driven Approach," *ACS Appl. Mater. Interfaces* (2022): *14*, 31203.

[6]	H. Sun, P. Ren, J. R. Fried, "The COMPASS force field: parameterization and validation for phosphazenes," *Comput. Theor. Polym. Sci.* (1998): *8*, 229.

[7]	H. Sun, "COMPASS:  An ab Initio Force-Field Optimized for Condensed-Phase ApplicationsOverview with Details on Alkane and Benzene Compounds," *J. Phys. Chem. B* (1998): *102*, 7338.

[8]	T. F. Willems, C. H. Rycroft, M. Kazi, et al., "Algorithms and tools for high-throughput geometry-based analysis of crystalline porous materials," *Micropor. Mesopor. Mat.* (2012): *149*, 134.

[9]	M. Pinheiro, R. L. Martin, C. H. Rycroft, et al., "Characterization and comparison of pore landscapes in crystalline porous materials," *J Mol. Graph. Model.* (2013): *44*, 208.

[10]	D. Ongari, P. G. Boyd, S. Barthel, et al., "Accurate Characterization of the Pore Volume in Microporous Crystalline Materials," *Langmuir* (2017): *33*, 14529.

[11]	T. H. Lee, M. Balcik, Z. Ali, et al., "Microporous polyimine membranes for efficient separation of liquid hydrocarbon mixtures," *Science* (2025): *388*, 839.

[12]	J. R. Perilla, B. C. Goh, C. K. Cassidy, et al., "Molecular dynamics simulations of large macromolecular complexes," *Curr. Opin. Struct. Biol.* (2015): *31*, 64.

[13]	D. C. Shi, H. Li, X. Yu, et al., "Intercrystalline channels at subnanometer scale for precise molecular nanofiltration," *J. Am. Chem. Soc.* (2023): *145*, 15848.

[14]	Z.-M. Xu, Z. Chen, X.-F. Zhong, et al., "Microenvironment Engineering of Conjugated Microporous Polymer Membranes Enabling Ultrahigh Solvent Permeability and Molecular Sieving," *J. Am. Chem. Soc* (2026): *148*, 2719.

[15]	B. Sengupta, Q. B. Dong, R. Khadka, et al., "Carbon-doped metal oxide interfacial nanofilms for ultrafast and precise separation of molecules," *Science* (2023): *381*, 1098.

[16]	K. Y. Wang, T. Matsuura, T.-S. Chung, et al., "The effects of flow angle and shear rate within the spinneret on the separation performance of poly(ethersulfone) (PES) ultrafiltration hollow fiber membranes," *J. Membr. Sci.* (2004): *240*, 67.

[17]	S.-L. Li, G. Chang, Y. Huang, et al., "2,2′-Biphenol-based Ultrathin Microporous Nanofilms for Highly Efficient Molecular Sieving Separation," *Angew. Chem. Int. Ed.* (2022): *61*, e202212816.

[18]	Z. Jiang, R. Dong, A. M. Evans, et al., "Aligned macrocycle pores in ultrathin films for accurate molecular sieving," *Nature* (2022): *609*, 58.

[19]	J. T. Liu, D. Hua, Y. Zhang, et al., "Precise Molecular Sieving Architectures with Janus Pathways for Both Polar and Nonpolar Molecules," *Adv. Mater.* (2018): *30,* 1705933.

[20]	T. F. Huang, T. Puspasari, S. P. Nunes, et al., "Ultrathin 2D-layered cyclodextrin membranes for high- performance organic solvent nanofiltration," *Adv. Funct. Mater.* (2020): *30*, 1906797.

[21]	T. Huang, B. A. Moosa, P. Hoang, et al., "Molecularly-porous ultrathin membranes for highly selective organic solvent nanofiltration," *Nat. Commun.* (2020): *11*, 5882.

[22]	Y. Xu, F. You, H. Sun, et al., "Realizing Mussel-Inspired Polydopamine Selective Layer with Strong Solvent Resistance in Nanofiltration toward Sustainable Reclamation," *ACS Sustainable Chem. Eng.* (2017): *5*, 5520.

[23]	B. Liang, H. Wang, X. H. Shi, et al., "Microporous membranes comprising conjugated polymers with rigid backbones enable ultrafast organic-solvent nanofiltration," *Nat. Chem.* (2018): *10*, 961.

[24]	Y. Jin, A. Zhang, G. Dong, et al., "Ultrathin membranes comprising polymers of intrinsic microporosity oligomers for high-performance organic solvent nanofiltration," *J. Membr. Sci.* (2025): *714*, 123436.

[25]	F. Alduraiei, S. Kumar, J. T. Liu, et al., "Rapid fabrication of fluorinated covalent organic polymer membranes for organic solvent nanofiltration," *J. Membr. Sci.* (2022): *648*, 120345.

[26]	F. Alduraiei, P. Manchanda, B. Pulido, et al., "Fluorinated thin-film composite membranes for nonpolar organic solvent nanofiltration," *Sep. Purif. Technol.* (2021): *279,* 119777.

[27]	S. Li, S. Du, S. Liu, et al., "Ultra-smooth and ultra-thin polyamide thin film nanocomposite membranes incorporated with functionalized MoS_2_ nanosheets for high performance organic solvent nanofiltration," *Sep. Purif. Technol.* (2022): *291*, 120937.

[28]	M.-B. Wu, F. Yang, J. Yang, et al., "Lysozyme Membranes Promoted by Hydrophobic Substrates for Ultrafast and Precise Organic Solvent Nanofiltration," *Nano Lett.* (2020): *20*, 8760.

[29]	X. He, H. Sin, B. Liang, et al., "Controlling the selectivity of conjugated microporous polymer membrane for efficient organic solvent nanofiltration," *Adv. Funct. Mater.* (2019): *29*, 1900134.

[30]	J.-B. Li, C.-Y. Zhu, B.-B. Guo, et al., "Ultrahigh-permeance polyamide thin-film composite membranes enabled by interfacial polymerization on a macro-porous substrate toward organic solvent nanofiltration," *J. Membr. Sci.* (2024): *693*, 122342.

[31]	S. Y. Li, R. J. Dong, V. E. Musteata, et al., "Hydrophobic polyamide nanofilms provide rapid transport for crude oil separation," *Science* (2022): *377*, 1555.

[32]	Z. Zhou, X. Li, D. Guo, et al., "Electropolymerization of robust conjugated microporous polymer membranes for rapid solvent transport and narrow molecular sieving," *Nat. Commun.* (2020): *11*, 5323.
